# Detection and validation of QTLs for green stem disorder of soybean (*Glycine max* (L.) Merr.)

**DOI:** 10.1270/jsbbs.23042

**Published:** 2024-03-23

**Authors:** Daisuke Ogata, Fumio Taguchi-Shiobara, Osamu Uchikawa, Masayuki Miyazaki, Yushi Ishibashi

**Affiliations:** 1 Fukuoka Agriculture and Forestry Research Center, 587 Yoshiki, Chikushino, Fukuoka 818-8549, Japan; 2 Graduate School of Bioresource and Bioenvironment Science, Kyushu University, 744 Motooka, Nishi, Fukuoka, Fukuoka 819-0395, Japan; 3 Institute of Crop Science, NARO, 2-1-2 Kannondai, Tsukuba, Ibaraki 305-8518, Japan

**Keywords:** soybean, green stem disorder, QTL, DNA marker, *Glycine max* (L.) Merr.

## Abstract

In mechanically harvested soybean, green stem disorder (GSD) is an undesirable trait that causes green-stained seeds, which are graded lower in Japan. To obtain DNA markers for reduced GSD, we conducted a quantitative trait locus (QTL) analysis for 2 years using F_4_ and F_5_ lines from a cross between ‘Suzuotome’ (less GSD) and ‘Fukuyutaka’ (more GSD). We validated the effect of a detected QTL for GSD by first identifying F_4_ or F_5_ plants in which one or more markers in the QTL region were heterozygous. The F_5_ or F_6_ progeny of each plant was used to form a pair consisting of two groups in which the QTL region was homozygous for either the ‘Suzuotome’ or ‘Fukuyutaka’ allele in a similar genetic background, and the two groups within each pair were compared for GSD. Over 3 years of testing, the ‘Suzuotome’ allele of a QTL on chromosome 6 was found to reduce the level of GSD. This novel QTL was mapped to the region around DNA marker W06_0130, and was not closely linked to QTLs for important agronomic traits including yield components. Using this marker, the low level of GSD from ‘Suzuotome’ could be conferred to ‘Fukuyutaka’ or other high-GSD cultivars.

## Introduction

Green stem disorder (GSD) in soybean (*Glycine max* (L.) Merr.) is a condition that causes the stems and leaves to stay green and retain moisture even after the pods have reached maturity. In Japan, GSD is a serious problem for soybean production. During machine harvesting, the seed coat surfaces are stained by green tissue fluid, and these stained seeds are graded as a lower class in the Japanese market (Morita *et al.* 2006, Ogiwara 2002). Thus, when many soybean plants are still green at harvest time, farmers have to remove those plants before harvest, which decreases yield and is laborious and time-consuming. To improve productivity, varieties without GSD are desirable.

The level of GSD in soybean is increased when the sink–source balance is disrupted and the source is excessive (Egli and Bruening 2006, Ogiwara 2002, Zheng 2005). Because of the effects of global warming in recent years, abnormal weather that disrupts the sink–source balance in soybean plants has occurred frequently, and the occurrence of GSD in soybean has increased and has been reported in some places in Japan (MAFF 2022). Since global warming is predicted to advance in the future (JMA 2017), the risk of unfavorable weather such as high temperature, drought, heavy rainfall and low sunshine during the soybean growing season, which reduces the number of pods and disrupts the sink–source balance, is likely to increase, and the occurrence of GSD in soybean may become a more serious problem.

Since varietal differences in the level of GSD have been reported (Hill *et al.* 2006, Matsumoto *et al.* 1986), it should be genetically possible to decrease the level of GSD in soybean. Further, the development of DNA markers linked to quantitative trait loci (QTLs) for GSD would enable more efficient breeding for a low-GSD trait.

The occurrence of GSD in the leading soybean variety ‘Fukuyutaka’ has become a problem in recent years in the Northern Kyushu area of Japan (Uchikawa *et al.* 2017). On the other hand, the soybean variety ‘Suzuotome’, which is also grown in Northern Kyushu, is reported to have a lower level of GSD than ‘Fukuyutaka’ and is a promising material for soybean breeding programs that are targeting GSD reduction (Ogata *et al.* 2021).

In this study, we conducted QTL analysis for GSD using F_4_ and F_5_ lines derived from a cross between ‘Suzuotome’ (less GSD) and ‘Fukuyutaka’ (more GSD). The effect of the detected QTL on GSD was validated using pairs of selected F_5_ or F_6_ plants derived from heterozygous F_4_ or F_5_ plants. This QTL was also tested for its effects on other agronomic traits such as flowering date and yield components.

## Materials and Methods

### Plant materials

A total of 186 F_4_ and F_5_ lines from a cross between ‘Suzuotome’ (Matsunaga *et al.* 2003) and ‘Fukuyutaka’ (Ohba *et al.* 1982) were developed by the single-seed descent method. ‘Suzuotome’, a variety used for natto (fermented soybeans) processing, shows less GSD. By contrast, the leading variety for tofu processing, ‘Fukuyutaka’, often shows more GSD (Ogata *et al.* 2021). The F_4_ and F_5_ lines were used for field experiments in 2018 and 2019, respectively. The genotypes of 186 F_4_ lines, excluding markers with heterogeneous genotypes, were used to construct a linkage map, which was used for QTL analysis.

To evaluate the effects of the QTL region detected on chromosome 6 (see Results), we compared genetically similar pairs of lines differing for QTL alleles in this region. To construct the pairs, three F_4_ plants (#192, #83 and #40) and two F_5_ plants (#54 and #75) that were heterozygous for one or more markers in the QTL region on chromosome 6 were selected in 2018 and 2019, respectively. Out of the F_5_ or F_6_ progeny of these selections, plants in which the QTL region was homozygous for either the ‘Suzuotome’ or ‘Fukuyutaka’ allele were selected and grouped into a pair of lines, one for each parental allele. For field experiments, we tested the three pairs derived from F_4_ plants #192, #83 and #40 from 2019 to 2021. In addition, the pair derived from F_5_ plant #54 was tested in 2020 and 2021, and the pair derived from F_5_ plant #75 was tested in 2021.

### Growth conditions

Plants were grown in fields with sandy loam soil at the Fukuoka Agriculture and Forestry Research Center (33°31ʹN, 130°30ʹN) in Japan for 4 years from 2018 to 2021. Inter-row and inter-hill intervals were 0.7 m and 0.1 m, respectively. Three seeds were sown in every hill, and plants were thinned to one plant per hill after primary leaf expansion. Fertilizer was not applied, and herbicides were sprayed on the ground immediately after sowing. Inter-tillage and earthing-up were performed at approximately the 5-trifoliate-leaf stage. Insecticides, fungicides and herbicides were sprayed during the growing season as appropriate.

The F_4_ and F_5_ lines were sown on 12 July in both 2018 and 2019. Each line was planted in a 1.2-m row without replication. Pairs of selected F_5_ and F_6_ plants were sown on 9 July in 2019, on 7 July in 2020, and on 14 and 19 July in 2021. Each line within a pair was planted in a 1.8-m row with 2 or 3 replications in 2019, 4 replications in 2020 and 5 replications in 2021.

In addition, to consider the relationship between the GSD level in pairs of selected F_5_ and F_6_ plants conducted from 2019 to 2021 and the weather environment, we used the data on the average temperature and precipitation of Dazaifu City, Fukuoka Prefecture, close to Fukuoka Agriculture and Forestry Research Center, as published by the Japan Meteorological Agency (https://www.data.jma.go.jp/stats/etrn/index.php).

### Evaluation of GSD level and agronomic traits

The level of GSD for each plant examined was evaluated by visual inspection at the maturity date in the experimental fields. We referred the GSD index (Takahashi 2013) to measure GSD, which was scored at 11 levels (0, 0.5, 1, 1.5, 2, 2.5, 3, 3.5, 4, 4.5, 5) with respect to stem and leaf conditions as follows: 0: leaflets and leaf stems had dropped off, and the stem was dry and brown; 1: leaflets and leaf stems had dropped off, and the stem remained a little green; 2: leaflets and leaf stems had dropped off, and the stem remained vivid green; 3: leaflets had dropped off, some of the leaf stems remained, and the stem remained vivid green; 4: most leaflets had dropped off, some of the leaf stems remained, and the stem remained vivid green; 5: most leaflets remained, and the stem remained vivid green. The assignment of the GSD index to each line was based on the most common level observed for individual plants of the line and was increased 0.5 when a plant displaying a higher level than the most common one was included.

In addition to GSD index, F_5_ pairs derived from F_4_ plants #192, #83 and #40 were also evaluated for seven agronomic traits: flowering date, maturity date, main stem length, seed pod number (/m^2^), grain number (/m^2^), 100-seed weight and total seed weight (kg/a). The flowering date was defined as the date of first anthesis (R1; Fehr and Caviness 1977) for 40–50% of the plants in a plot. The maturity date was defined as the date on which 95% of the pods had reached mature pod color (R8; Fehr and Caviness 1977) for 80% of the plants in a plot.

The significance of the results was evaluated with the software Statcel 4 (OMS Inc., Tokorozawa, Japan) using Spearman’s correlation coefficient by rank test, Kruskal–Wallis test and single-factor ANOVA.

### Genotyping and QTL analysis

Total DNA was extracted from leaves of F_4_ plants and cotyledons of their seeds (F_5_ generation) by using a BioSprint 96 automatic DNA isolation system (Qiagen K. K., Tokyo, Japan). Polymerase chain reaction (PCR) amplification was performed using a Qiagen Multiplex PCR Kit (Qiagen) at 95°C for 15 min; followed by 35 cycles of 30 s at 95°C, 90 s at 50°C and 90 s at 60°C; and a final 60°C for 30 min. The lengths of PCR products were measured by using a 3730xl DNA Analyzer (Thermo Fisher Scientific), and allele calling and binning were performed with GeneMapper 5.0 software (Thermo Fisher Scientific).

SSR markers described in Fujii *et al.* (2018) were used for map construction and genotyping. The marker names in Fujii *et al.* (2018) are in the format ‘WGSP xx_xxxx’, abbreviated here as ‘Wxx_xxxx’. A linkage map was constructed using 186 F_4_ lines and 171 markers in MAPMAKER/EXP 3.0 software (Lander *et al.* 1987). The genetic distance was estimated by using the software’s Kosambi map function (Kosambi 1944) in the ‘ri’ setting. When genetic distance was more than 40 cM, markers in a chromosome were grouped into two linkage groups (which occurred for chromosomes 10 and 15). The total lengths of the linkage maps for all 20 chromosomes were 1985.0 cM, and the average marker interval was 13.3 cM. This linkage map of F_4_ lines, in which heterozygous alleles were regarded as missing values, was used for the QTL analysis.

Composite interval mapping (CIM) analyses were performed to detect potential QTL positions using the R/qtl software ver. 4.2.2 (https://www.rqtl.org; Broman and Sen 2009). Genotype data were converted by ‘convert2riself’. A coarse grid (5 cM) was used for the sake of computational speed. The LOD score curves were obtained by using the ‘cim’ function. The CIM threshold was based on the results of 1,000 permutations at a 5% significance level (Churchill and Doerge 1994). To obtain estimates of QTL effects, multiple-QTL mapping was performed using ‘sim.geno’, ‘makeqtl’, ‘refineqtl’ and ‘fitqtl’ functions (Broman and Sen 2009). To identify the interactions between QTLs, the ‘addint’ function was used (Broman and Sen 2009).

## Results

### Detection of QTLs affecting GSD

**Fig. 1.**
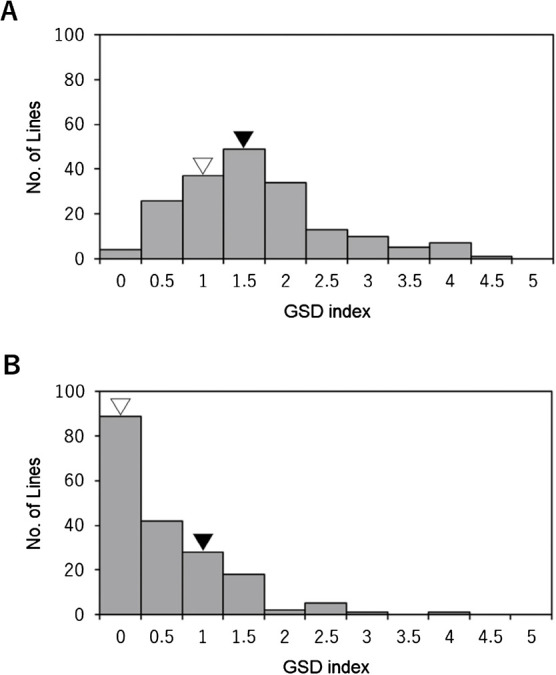
Frequency distribution of the average value of green stem disorder (GSD) in F_4_ lines in 2018 (A) and F_5_ lines in 2019 (B). ▽: ‛Suzuotome’; ▼: ‛Fukuyutaka’.

**Fig. 2.**
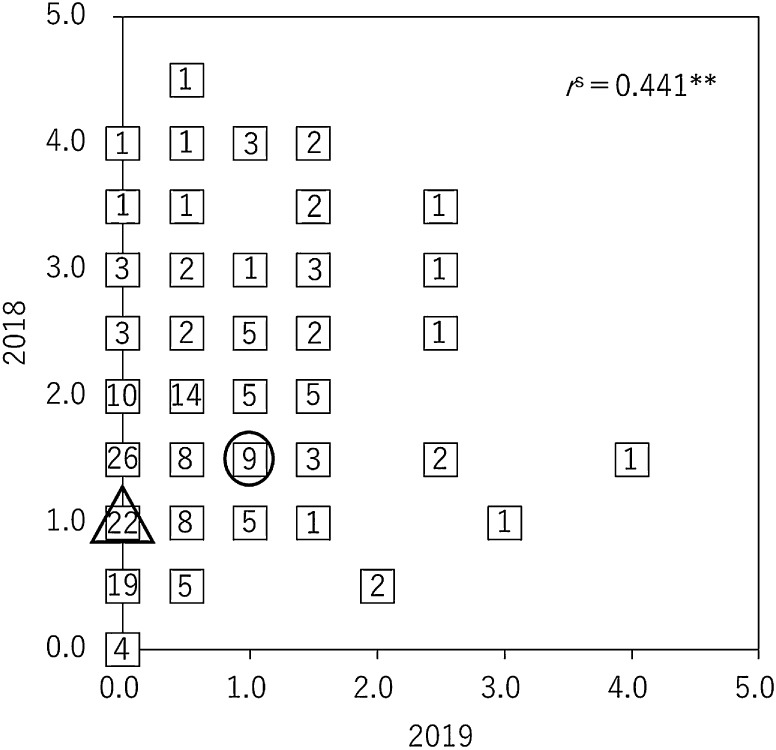
Scatter plot of the green stem disorder (GSD) indexes of F_4_ lines in 2018 (vertical) and F_5_ lines in 2019 (horizontal). △: ‘Suzuotome’, 〇: ‘Fukuyutaka’. The number in each square indicates the number of overlapping samples. **: significant at the 1% level using Spearman’s correlation coefficient by rank test.

In 2018, the GSD index of ‘Suzuotome’ and ‘Fukuyutaka’ was 1 and 1.5, respectively, and the GSD index in the 186 F_4_ lines was distributed between 0 and 4.5 (Fig. 1A). In 2019, the GSD index of ‘Suzuotome’ and ‘Fukuyutaka’ was 0 and 1, respectively, and the GSD index in the 186 F_5_ lines was distributed between 0 and 4 (Fig. 1B). The correlation of the GSD indexes between F_4_ lines in 2018 and F_5_ lines in 2019 was significant at the 1% level (*r*^s^ = 0.441; Fig. 2).

**Fig. 3.**
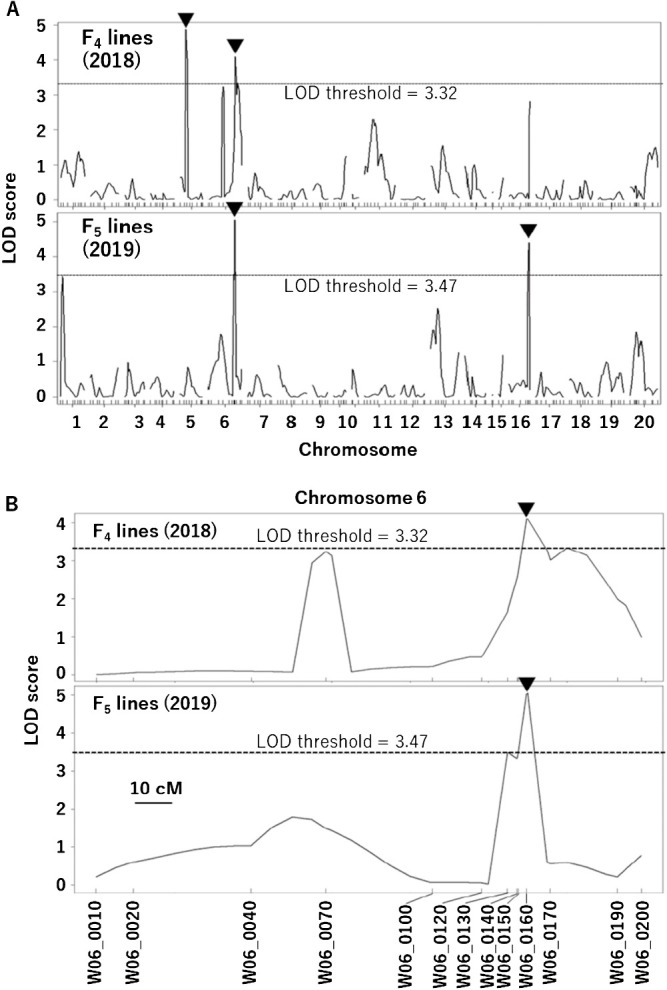
LOD score curves for the QTLs for green stem disorder (GSD) of soybean in F_4_ lines (2018) and F_5_ lines (2019) obtained by CIM. (A) Triangles show LOD score peaks that were above the threshold. The *x* axes represent the linkage map of F_4_ lines, with DNA marker positions shown as vertical lines. Dotted horizontal lines are LOD thresholds. (B) Potential positions of QTLs on chromosome 6. Triangles show LOD score peaks that were above the threshold. Vertical lines indicate the relative genetic positions of DNA markers.

Through CIM, four potential QTLs for GSD were detected: one each on chromosomes 5 and 6 in 2018, and one each on chromosomes 6 and 16 in 2019 (Fig. 3A). Further multiple-QTL mapping supported all of these putative QTLs except for the one on chromosome 5 detected in 2018 (Table 1). When the locations of putative QTLs were refined in a multiple-QTL model using the ‘refineqtl’ function, positions of QTLs were not moved except for the putative QTL on chromosome 6 detected in 2018, which was adjusted from position 110 cM (Fig. 3B) to 125 cM (Table 1). Among the three putative QTLs supported by multiple-QTL mapping, QTLs on chromosome 6 were detected in both years, and for each one the ‘Suzuotome’ allele reduced the GSD index. Although the two QTL peaks on chromosome 6 are close, it is not yet certain whether they are the same (Table 1). In 2019, LOD peak position 110 cM on chromosome 6, which nearest marker was W06_0160, explained for 10.4% of total variance (Table 1). On the other hand, in the QTL on chromosome 16 detected in 2019, the ‘Fukuyutaka’ allele reduced the GSD index (Table 1). Assuming full model (Q1 + Q2 + Q1*Q2), interaction between the two putative QTLs on chromosome 6 and 16 detected in 2019 was not significant (P value (F) = 0.1).

### Validation of the putative QTL for GSD

**Fig. 4.**
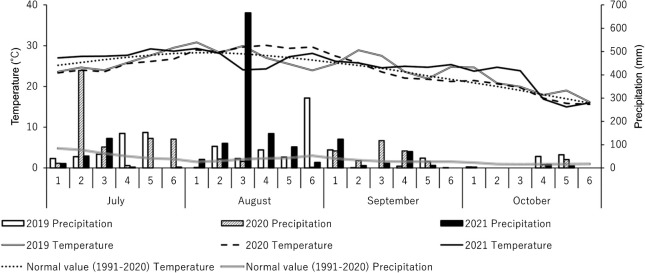
Average temperature and precipitation from July to October in 2019, 2020 and 2021. 1, 2, 3, 4, 5 and 6 in the diagram indicate 1st–5th, 6th–10th, 11th–15th, 16th–20th, 21st–25th and 26th–, respectively.

The average temperature and precipitation during the soybean growing period in 2019, 2020 and 2021 are shown in Fig. 4. The average temperature from 11 August to 5 September, which corresponded to the period before and after the flowering day, was 26.4°C (0.6°C lower than normal) in 2019, 29.3°C (2.2°C higher than normal) in 2020 and 26.0°C (1.0°C lower than normal) in 2021. Similarly, the precipitation amount from 11 August to 5 September was 544 mm (253% compared to normal) in 2019, 104 mm (48% compared to normal) in 2020 and 1049 mm (487% compared to normal) in 2021.

**Fig. 5.**
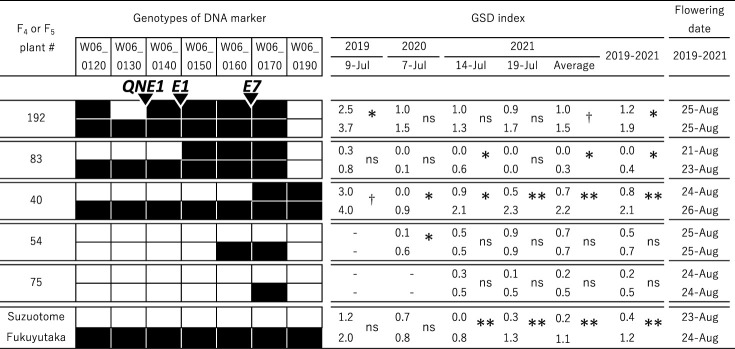
Comparisons of green stem disorder (GSD) index within pairs of selected F_5_ or F_6_ plants; each pair was derived from a F_4_ or F_5_ plant in which a part of the QTL region on chromosome 6 was heterozygous. For each marker, white and black boxes indicate the ‘Suzuotome’ allele and the ‘Fukuyutaka’ allele, respectively. The locations of flowering time genes, E1, E7 and QNE1, are shown for references. Note that QTL analysis for flowering time was not performed. **, * and † indicate significance at 1%, 5% and 10% levels using Kruskal–Wallis test; ns, not significant.

To validate the effect of the putative quantitative trait loci (QTLs) on chromosome 6 on the level of GSD, we developed five pairs of F_5_ or F_6_ lines from F_4_ plants #192, #83 and #40 and F_5_ plants #54 and #75, in which one or more genomic regions between markers W06_0120 and W06_0170 on chromosome 6 were found to segregate (Figs. 3B, 5).

The GSD indexes and the genotypes of the QTL region for each of the five pairs are shown in Fig. 5. The average GSD index over 3 years (2019–2021) for ‘Suzuotome’ was significantly lower than that for ‘Fukuyutaka’, although single-year differences were not always significant. In three pairs derived from F_4_ plants #192, #83 and #40, which all segregated for a genomic region around W06_0130, the average GSD indexes (2019–2021) were significantly lower for the ‘Suzuotome’ allele (shown as white) than for the ‘Fukuyutaka’ allele (shown as black). On the other hand, in 2021 when all five pairs from #192, #83, #40, #54 and #75 were cultivated, although the average GSD indexes in three pairs from #192, #83 and #40 were significantly different between the ‘Suzuotome’ and ‘Fukuyutaka’ allele, those in two pairs from #54 and #75, which have a common segregating genomic region around W06_0170, were not significantly different between the ‘Suzuotome’ and ‘Fukuyutaka’ allele.

### Effect of the QTL for GSD on agronomic traits

To examine the effect of the chromosome 6 QTL region on agronomic traits, three pairs derived from F_4_ plants #192, #83 and #40 were evaluated for seven traits: flowering date, maturity date, main stem length, seed pod number, grain number, 100-seed weight and total seed weight (Table 2).

The phenotypes of the parents, ‘Suzuotome’ and ‘Fukuyutaka’, are shown at the bottom of Table 2. The flowering date and maturity date of ‘Suzuotome’ were 1 day and 7 days earlier, respectively, than those of ‘Fukuyutaka’. Compared to ‘Fukuyutaka’, ‘Suzuotome’ had a longer main stem length, more pods and grains, a smaller 100-seed weight and a lower yield.

In the pair derived from F_4_ plant #192, none of the seven agronomic traits of the plants carrying the ‘Suzuotome’ allele were significantly different from those carrying the ‘Fukuyutaka’ allele (Table 2, top). In the pairs from #83 and #40 (Table 2, middle), the flowering date and maturity date of the plants carrying the ‘Suzuotome’ allele were earlier and the main stem length was significantly shorter than for those carrying the ‘Fukuyutaka’ allele. In the pair from #83, all yield components except for 100-seed weight were significantly lower in plants carrying the ‘Suzuotome’ allele than in those carrying the ‘Fukuyutaka’ allele. In the pair from #40, the seed pod number of plants carrying the ‘Suzuotome’ allele was significantly lower than in plants carrying the ‘Fukuyutaka’ allele. In the pair from #192, no significant difference was observed in agronomic traits (Table 2), and the average GSD index over 3 years of ‘Fukuyutaka’ allele was higher than that of ‘Suzuotome’ allele (Fig. 5). These results indicate that the QTL for GSD is not closely linked to QTLs for important agronomic traits.

## Discussion

In this study, we detected a QTL on chromosome 6 that could be used to breed the low-GSD trait from ‘Suzuotome’ into ‘Fukuyutaka’. The GSD index in 186 F_4_ and F_5_ lines was distributed between 0 and 4.5, and between 0 and 4, respectively (Fig. 1). The correlation of the GSD indexes between F_4_ lines in 2018 and F_5_ lines in 2019 was significant (Fig. 2). These findings indicate that the level of GSD is under genetic control, as described by Matsumoto *et al.* (1986) and Hill *et al.* (2006). We performed QTL analysis for GSD using F_4_ lines in 2018 and F_5_ lines in 2019. The QTL on chromosome 6 that reduced the GSD level when it was the ‘Suzuotome’ allele was detected in both 2018 and 2019, and the QTL on chromosome 16 that reduced the GSD level when it was the ‘Fukuyutaka’ allele was detected in 2019 (Fig. 3, Table 1). The QTLs that reduced the GSD level were found in both the ‘Suzuotome’ and ‘Fukuyutaka’ allele, and this was suggesting the reason for the transgressive segregation of the GSD level in Fig. 1. We also verified the effect of the QTL on chromosome 6 detected in both years using pairs of selected F_5_ or F_6_ plants, each pair derived from an F_4_ or F_5_ plant in which one or more markers in the QTL region were heterozygous (Fig. 5).

We designed these experiments to shorten the time required as much as possible. When evaluating phenotypes repeatedly, RILs provide more information than F_4_ or F_5_ lines; this is because the resolution power should be larger for RILs owing to the higher frequency of fixed genomic regions than in F_4_ or F_5_ lines. QTL analysis using RILs would be more reliable than that using F_4_ lines. However, QTL of larger effect could be detected even in F_4_ lines, and existence of the QTL would be verified using F_5_ lines derived from the same F_4_ plant that were heterozygous for the QTL region since such F_5_ lines would share 87.5% genetic background which was fixed in the F_4_ plant. Actually Glover *et al.* (2004) successfully detected QTL conferring soybean cyst nematode resistance using F_4_ lines, and confirmed the existence of the QTL using lines derived from F_4_ plant that were heterozygous for the QTL region. Thus, we used F_4_ and F_5_ lines and successfully detected one QTL region with a large effect even in F_4_ lines, allowing us to save time. We selected plants homozygous for each parental allele from families of F_5_ or F_6_ plants (F_4_- or F_5_-derived lines) in which the background was nearly fixed. For each heterozygous F_4_ or F_5_ plant, selected F_5_ or F_6_ progeny were grouped into a pair of lines according to genotype (i.e., homozygous for one parental allele or the other) and used for phenotypic evaluation without obtaining descendant lines. Again, this enabled us to shorten the period of our study. The ability to evaluate phenotypes without obtaining descendant lines was also reported by Tuinstra *et al.* (1997), who verified QTLs for seed weight sorghum.

To validate the effect of the detected QTL on chromosome 6 on the level of GSD, we developed five pairs from F_4_ and F_5_ plants, in which one or more genomic regions between markers W06_0120 and W06_0170 on chromosome 6 were found to segregate. As a result, in three pairs derived from F_4_ plants #192, #83 and #40, which all segregated for a genomic region around W06_0130, the average GSD index (2019-2021) was significantly lower for the ‘Suzuotome’ allele than for the ‘Fukuyutaka’ allele (Fig. 5). In the pair from #83, ‘Fukuyutaka’ allele showed lower GSD index than ‘Suzuotome’, implying that this pair had unknown QTL in their background. On the other hand, in 2021 when all five pairs were cultivated, significant difference in the GSD index was not detected between ‘Suzuotome’ and ‘Fukuyutaka’ allele in two pairs from #54 and #75, which segregated for a genomic region between W06_0160 and W06_0170, or around W06_0170, although that in three pairs from #192, #83 and #40 was detected (Fig. 5). These suggested that the location of the QTL for GSD on chromosome 6 was the region around W06_0130. In previous studies, Pierce *et al.* (1984) and Fujii *et al.* (2015) reported that QTLs associated with GSD were related to the indeterminate growth QTL on chromosome 19, and Yamada *et al.* (2014) reported 3 QTLs for GSD: *qGSD1* on chromosome 12, *qGSD2* on chromosome 13 and *qGSD3* on chromosome 19. Thus, the GSD QTL detected around W06_0130 on chromosome 6 in this study is novel.

We could not disprove that other QTLs for GSD might be in the region around W06_0120 and/or between W06_0140 and W06_0170. Since percentage of total phenotypic variance of QTL was larger in 2019 than in 2018 (Table 1), experimental errors might be smaller in 2019 than in 2018. In 2019, peak position of the QTL was 110 cM on chromosome 6 and the nearest marker was W06_0160 (109.7 cM) (Table 1). LOD score at W06_0130 (104.8 cM) showed small peak but its score was just below the threshold level (=3.47) (Fig. 3B). We detected the QTL around W06_0130, but another QTL of larger effect might exist in the region between W06_0150 and W06_0160. The result that the peak of LOD score curve was around W06_160 in both 2018 and 2019 (Fig. 3) also support this idea. More pairs that cover these regions are needed to clarify whether QTLs for GSD are present there. Furthermore, as discussed in the next paragraph, the occurrence of GSD is also influenced by the environment, so further tests under many environmental conditions including multi-year cultivation are needed.

Uchikawa *et al.* (2017) reported that the occurrence of GSD in soybean plants was affected by environmental factors, such as reducing the number of pods. Additionally, it has been reported that heavy rainfall before and after the flowering reduced the number of pods (Uchikawa *et al.* 2003) and that deficient or excessive soil moisture around the flowering promoted the fall of the buds, the flowers and the pods (Fukui and Ojima 1951). It is difficult to stably judge the difference in the level of GSD among varieties in a single study because depending on environmental conditions there are years when GSD is not occurred regardless of whether characteristics with less or more GSD. Therefore, in this study we dealt with the problem by implementing multi-year or multi-seeding cultivation. In fact, under weather conditions before and after the flowering, the precipitation amount in 2019 and 2021 was higher than normal, indicating the excessive soil moisture, and that in 2020 was lower than normal, indicating the deficient soil moisture (Fig. 4). These environmental conditions may have more or less influenced the GSD level in five pairs derived from F_4_ and F_5_ plants. The results that single-year differences in the level of GSD between ‘Suzuotome’ and ‘Fukuyutaka’, and between the pairs derived from F_4_ plants #192, #83 and #40 were not always significant were thought to be due to the environmental influence.

On regarding the relationship between GSD and some agronomic traits, Harbach *et al.* (2016) showed that the level of GSD was correlated with yield, plant height, degree of lodging, etc. Uchikawa and Hirata (2012) showed that the flowering date of lines with more GSD was earlier, the main stem length was shorter, the grain number was smaller and the total seed weight was lower than those of lines without GSD. In the pair derived from F_4_ plant #192, the phenotypes of plants carrying the ‘Suzuotome’ allele and those carrying the ‘Fukuyutaka’ allele did not differ significantly for any of the seven agronomic traits tested (Table 2). This indicates that the QTL for GSD detected in this study is not closely linked to others associated with important agronomic traits such as yield components. On the other hand, in the pairs derived from F_4_ plants #83 and #40, the flowering date of plants carrying the ‘Suzuotome’ allele was two days earlier than for plants carrying the ‘Fukuyutaka’ allele (Table 2). The detected QTL regions on chromosome 6 included at least three loci which were associated with flowering-time: the E1, E7 and QNE1 loci (Abe *et al.* 2003, Cober and Voldeng 2001, Cregan *et al.* 1999, Molnar *et al.* 2003, Xia *et al.* 2022) (Fig. 5). As for E7 locus, ‘Suzuotome’ allele and ‘Fukuyutaka’ allele would be the same because flowering dates of the pairs from F_4_ plants #54 were the same. The alleles at QNE1 or E1 loci of ‘Suzuotome’ might not be the same as those of ‘Fukuyutaka’. Since flowering times of the pairs from F_4_ plants #83 or #40 were different, the regions which were covered by the pair would contribute to flowering-time in addition to the index of GSD. Decreased main stem length, seed pod number, grain number and total seed weight associated with the ‘Suzuotome’ allele in F_4_ plants #83 and #40 could also be attributed to the shorter vegetative growth period (Inoue 2006).

In conclusion, we detected a novel QTL on chromosome 6 that was associated with GSD level but not closely linked to QTLs controlling important agronomic traits including yield components. By using marker W06_0130, which was identified through QTL analysis and subsequent genetic analysis, it should be possible to introgress the low-GSD trait from ‘Suzuotome’ into leading variety ‘Fukuyutaka’. Other varieties with less GSD could also be developed in breeding programs.

## Author Contribution Statement

DO, OU and MM designed the research, performed the field experiments and bred the breeding lines. FT genotyped the breeding lines. DO, FT and YI analyzed the data and wrote the manuscript. All authors read and approved the manuscript.

## Figures and Tables

**Table 1.** Putative quantitative trait loci (QTLs) for green stem disorder (GSD) detected by ANOVA

Year	Chr.	Nearest DNA marker	Peak position (cM)	LOD*^a^*	AE*^b^*	PVE*^c^*	P-value*^d^*
2018	5	W05_0070	25	0.1	0.0	0.2	0.51 ns
	6	W06_0190	125*^e^*	2.5	0.2	5.9	8.07E-04**
2019	6	W06_0160	110	4.9	0.2	10.4	1.76E-05**
	16	W16_0090	85	5.0	–0.2	10.8	1.28E-05**

*^a^* LOD: logarithm of the odds. *^b^* AE: additive effect. A positive additive effect value indicates that the ‘Suzuotome’ allele decreases the level of GSD. *^c^* PVE: percentage of total phenotypic variance explained by each QTL. *^d^* ** indicates significance at 1% level using ANOVA; ns, not significant. *^e^* When the locations of putative QTLs were refined in a multiple-QTL model using the ‘refineqtl’ function, it was moved from position 110 cM to 125 cM.

**Table 2.** Seven agronomic traits*^a^* of three pairs derived from F_4_ plants #192, #83 and #40, which segregate for markers in the GSD QTL region on chromosome 6

F_4_ plant #	Allele	Flowering date	Maturity date	Main stem length (cm)	Seed pod number (/m^2^)	Grain number (/m^2^)	100-seed weight (g)	Total seed weight (kg/a)
192	Suzuotome	25-Aug	31-Oct	63	952	1529	19.3	29.3
	Fukuyutaka	25-Aug	31-Oct	63	942	1513	20.0	30.1
				ns	ns	ns	ns	ns
83	Suzuotome	21-Aug	27-Oct	62	1154	1813	17.4	31.2
	Fukuyutaka	23-Aug	28-Oct	70	1268	2101	17.4	36.3
				*	*	**	ns	**
40	Suzuotome	24-Aug	30-Oct	67	1021	1516	21.5	32.5
	Fukuyutaka	26-Aug	1-Nov	74	1049	1557	21.1	32.8
				*	*	ns	ns	ns
Suzuotome		23-Aug	25-Oct	66	1323	2245	11.3	25.2
Fukuyutaka		24-Aug	1-Nov	58	756	1195	27.7	33.2
				**	**	**	**	**

*^a^* Data for the three pairs derived from F_4_ plants represent the average of 3 years (2019 to 2021). For ‘Suzuotome’ and ‘Fukuyutaka’, the averages are for 2 years (2019 and 2021). *^b^* ** and * indicate significance at the 1% and 5% levels using single-factor ANOVA or Kruskal–Wallis test, respectively; ns, not significant.
